# Supplementary material for: The PreQ-20 TRIAL: A prospective cohort study of the oncologic safety, quality of life and cosmetic outcomes of patients undergoing prepectoral breast reconstruction
Source: PLoS One. 2022 Jul 14;17(7):e0269426. doi: 10.1371/journal.pone.0269426 (PMC9282436; doi:10.1371/journal.pone.0269426)
Supplement: S1 File — (DOCX) [file pone.0269426.s002.docx]

PREPECTORAL RECONSTRUCTION AFTER ONCOLOGICAL AND RISK REDUCTION MASTECTOMY

PROSPECTIVE STUDY FOR THE ASSESSMENT OF YOUR SAFETY, QUALITY OF LIFE AND COSMETIC SEQUELS

Promoter Code: PreQ-20

Registration Code: 2020/295

**Promoter / Principal Investigator:**

BENIGNO ACEA NEBRIL

Breast Unit. General Surgery and Digestive System Service.

University Hospital Complex of A Coruña.

Version 1 of May 14, 2020

**OPINION OF THE A CORUÑA-FERROL RESEARCH ETHICS COMMITTEE**

Natalia Cal Purriños, secretary of the research ethics committee of A Coruña - Ferrol

**CERTIFIES:**

That this committee evaluated the study on 07/20/20:

**Title:** Prepectoral reconstruction after oncological and risk reduction mastectomy. Prospective study for the assessment of your safety, quality of life and cosmetic sequels

**Version:** V1

**Promoter:** Benigno Acea Nebril

**Investigator:** Benigno Acea Nebril

and that this committee, taking into consideration the relevance of the study, the available knowledge, the ethical, methodological and legal requirements applicable to research studies with human beings, their samples or registration and the committee's standard work procedures, issues a **FAVORABLE** opinion to carry out the aforementioned study.

**AND ESTABLISHES THAT:**

1. The Territorial Ethics Committee of the A Coruña-Ferrol Investigation complies with current legal requirements

2. The current composition of the A Coruña-Ferrol Territorial Research Ethics Committee is:

**Carmen Mella Pérez (President).** Medical Specialist in Internal Medicine. Ferrol Integrated Management Area.

**Angel Lopez-Silvarrey Varela (Vice President).** Specialist in Pediatrics. Integrated Management Area A Coruña.

**Natalia Cal Purriños (Secretary).** Degree in Law. "Professor Novoa Santos" Foundation. A Coruña.

**Sonia Pértega Díaz (Deputy Secretary).** Mathematics. Integrated Management Area A Coruña.

**Juana Mº Cruz del Rio.** Social Worker. Department of Health.

**María Ángeles Freire Fojo.** Pharmaceutical. Specialist in Hospital Pharmacy. Ferrol Integrated Management Area.

**González Lorenzo Portal.** Specialist in Family and Community Medicine. Integrated management area Ferrol

**Isaac Martínez Bendayán.** Cardiology Specialist Physician. Integrated Management Area A Coruña.

**Maria Otero Santiago.** Specialist in Preventive Medicine and Public Health. Integrated Management Area A Coruña.

**Alejandro Pazos Sierra.** Doctor. University of A Coruña.

**Gonzalo Peña Pérez.** Cardiology Specialist Physician. San Rafael Hospital. A Coruña.

**Carlos Rodríguez Moreno.** Specialist in Clinical Pharmacology. Santiago Integrated Management Area.

**Jose Mº Rumbo Prieto.** Diploma in Nursing. Ferrol Integrated Management Area

**María Isabel Sastre Gervás.** Pharmaceutical Primary Care. Integrated Management Area A Coruña.

For the record, where appropriate, and at the request of the appropriate person, in A Coruña.

The Secretary of the Territorial Committee of Research Ethics of A Coruña - Ferrol,

**Natalia Cal Purriños**

**CONFORMITY OF THE CENTER'S ADDRESS**

Mr. José Manuel Vázquez Rodríguez, Coordinator of Teaching and R + D + i of the Integrated Management Department of A Coruña.

**CERTIFIES**

Who knows the proposal made by the Promoter and Principal Investigator, Mr. Benigno Acea Nebril, from the General Surgery and Digestive System Service. Breast Unit, of the Health Area of ​​A Coruña and Cee, so that the clinical study entitled “: Prepectoral reconstruction after oncological and risk reduction mastectomy. Prospective study for the assessment of your safety, quality of life and cosmetic sequels” and Protocol code PreQ-20

That he accepts the realization of a clinical study in this Center, which has a **FAVORABLE** opinion from the Research Ethics Committee of A Coruña - Ferrol dated 06-22-2020 and Registration Code: 2020/295

The access, collection and processing of personal data of the subjects included in the study will be carried out in strict compliance with current legislation on the matter: Law 41/2002, of November 14, basic regulating the autonomy of the patient and rights and obligations regarding information and clinical documentation; Law 3/2005 of Galicia on regulations governing informed consent and the medical history of patients, Organic Law 3/2018, of December 5, on the Protection of Personal Data and guarantee of digital rights and Regulation (EU) 2016 / 679 of the European Parliament and of the Council, of April 27, 2016, regarding the Protection of natural persons with regard to the processing of personal data and the free circulation of these data.

A Coruña, July 10, 2020

**INDEX**

1. Summary

2. General information

3. Project justification

4. Objectives

5. Type of study

6. Patients and method

7. Ethical-legal aspects

8. Financing

9. Commitment of the researcher

10. Commitment to publication

11. Bibliography

12. Information sheet for the adult participant

13. Informed consent document

14. Witness informed consent document for participation in a research study (for cases where the participant cannot read / write)

**1. Summary**

Currently, mastectomy is a necessary surgical procedure in two clinical settings. The first refers to the oncological field where at least 30% of patients with breast cancer will require complete removal of this organ to guarantee locoregional control of their disease. The second focuses on the area of ​​high risk for the development of breast cancer, generally associated with the hereditary syndrome for breast and ovarian cancer, and which allows some high-risk women to decide on a risk reduction mastectomy (also called prophylactic) to prevent future development of breast tumors. In both circumstances, the removal of the breast can be complemented with a reconstruction procedure during the same surgical act (immediate breast reconstruction), which avoids the negative impact on the body image in women. Different studies (1-3) have shown that planning an immediate reconstruction after mastectomy improves the satisfaction and quality of life of women and therefore has become an option that should be discussed with the patient during the decision-making process.

During the last years the techniques of mastectomy and breast reconstruction have evolved towards less aggressive procedures and with a greater preservation of the anatomical elements. Thus, mastectomies aimed at immediate reconstruction have evolved into procedures that preserve the skin envelope of the breast, the inframammary groove and, on occasions, the areola-nipple complex. On the other hand, the reconstructive procedures have been oriented to the placement of subcutaneous breast implants and above the pectoralis major muscle, the so-called prepectoral reconstruction, which has allowed less postoperative morbidity and a more natural breast reconstruction. Several studies (4-6) published during the last 5 years have shown that the association of a skin-sparing mastectomy and a prepectoral reconstruction guarantees good results both in the oncological context and in reducing risk.

Despite these improvements and progress, some unknowns currently persist about different clinical aspects related to prepectoral reconstruction associated with a skin-sparing mastectomy. From a safety point of view, it is necessary to know the amount of residual breast tissue that persists after mastectomy, its predisposing factors and its evolution in the medium term. This circumstance is especially important in women operated on for high risk since they can develop breast cancer in the residual breast tissue. Furthermore, in patients treated for breast cancer, it is necessary to know the local relapse rate to establish the impact of this procedure on disease-free survival. From the perspective of women, it is important to know the impact of this procedure on satisfaction and quality of life through information reported by the patient herself. Therefore, the pre and postoperative evaluation of satisfaction and quality of life using PRO questionnaires, such as the BreastQ questionnaire, allows evaluating changes in this area after surgery. Finally, there is little information on the stability of this surgical technique during patient follow-up, as well as the need for new surgical interventions to solve the cosmetic sequelae (deformities, asymmetries) that occur over time. These uncertainties have encouraged us to carry out this prospective study in order to analyze the safety, satisfaction and cosmetic sequelae in patients treated in our breast unit by means of a prepectoral reconstruction after skin-sparing mastectomy.

**2. General information**

**2.1 Project title.**

PREPECTORAL RECONSTRUCTION AFTER ONCOLOGICAL AND RISK REDUCTION MASTECTOMY. PROSPECTIVE STUDY FOR THE ASSESSMENT OF YOUR SAFETY, QUALITY OF LIFE AND COSMETIC SEQUELS

**2.2 Protocol code.**

PreQ-20

**2.3 Promoter / Principal Investigator and collaborators.**

Promoter / Researcher: Benigno Acea Nebril. Breast Unit. General Surgery and Digestive System Service. University Hospital Complex of A Coruña.

Collaborating Researchers:

Lourdes García Jiménez. General Surgery and Digestive System Service. University Hospital Complex of A Coruña.

Alberto Bouzón Alejandro. Breast Unit. General Surgery and Digestive System Service. University Hospital Complex of A Coruña.

Carlota Díaz Carballada. Breast Unit. General Surgery and Digestive System Service. University Hospital Complex of A Coruña.

Carmen Cereijo Garea. Breast Unit. Nursing. University Hospital Complex of A Coruña.

Angela Iglesias López. Breast Unit. Radiology Service. University Hospital Complex of A Coruña.

Alejandra García Novoa. General Surgery Service. University Hospital Complex of A Coruña.

Pilar Casteleiro Roca. Plastic Surgery Service. University Hospital Complex of A Coruña.

**2.4 Centers in which the study is planned.**

Breast Unit. University Hospital Complex of A Coruña. Breast Units in Spain and Portugal with approval of this study by their research ethics committees

**3. JUSTIFICATION OF THE PROJECT**

Since 2013, breast units have experienced an increase in care for high-risk women as a consequence of the so-called “Jolie effect” that has led to greater social and professional awareness about the hereditary risk of suffering from breast cancer (7). This change has resulted in a greater dissemination of genetic studies and the opening of high-risk units in breast units, which has made it possible to identify a greater number of people with hereditary syndrome for breast and ovarian cancer, either through identification of a genetic mutation or verification of your family history. The final consequence of this phenomenon has been an increase in the care burden of breast units and especially an increase in risk reduction mastectomies and the need for breast reconstruction. These patients should be added to the patients who also require a mastectomy and reconstruction for breast cancer, which requires a surgical procedure that allows the safe removal of the breast, a reconstruction with low morbidity and a stable and satisfactory result over time. Skin-sparing mastectomy associated with prepectoral reconstruction has been proposed as a technical combination that can meet the aforementioned objectives.

However, its safety needs to be evaluated in terms of efficacy for glandular removal as well as for cancer control in women with breast cancer. Our unit has carried out two research projects to evaluate oncological safety in conservative (8) and oncoplastic (9,10) surgery for breast cancer, but we lack studies to evaluate safety in the mastectomies we perform. The first objective of this study will be the evaluation, in the short and medium term, of the safety of mastectomy in patients with breast cancer and in women who underwent risk reduction surgery. This will allow us to know the incidence of locoregional relapses and their risk factors.

Likewise, our unit carried out studies to evaluate satisfaction and quality of life in women who underwent conservative and oncoplastic breast surgery (11,12) as well as in patients reconstructed with an expander-prosthesis after mastectomy (13). On the contrary, we do not have information on the satisfaction and quality of life reported by the patients operated on using a prepectoral technique. The second objective of this study will be to evaluate the impact of this technical procedure on the satisfaction and quality of life reported by the patients through the BreastQ questionnaires. A secondary objective to this will be the identification of those factors that influence the final satisfaction of the patients.

Finally, our unit has described the cosmetic sequelae that occur in women with breast conservation during their follow-up (14) but we do not know what the stability over time of prepectoral reconstruction is. The third objective of this study will be the evaluation of the cosmetic sequelae of the reconstructed breast during the follow-up of the patient. A secondary objective to this will be the identification of risk factors for the appearance of these cosmetic sequelae.

**4. Objectives**

**4.1. Main objectives:**

1. To assess the safety of prepectoral reconstruction in women with high-risk breast cancer, in terms of residual tissue and local relapses.

2. To evaluate the satisfaction and quality of life after a prepectoral reconstruction in women with breast cancer and at high risk.

3. To evaluate the cosmetic sequelae after a prepectoral reconstruction in women with breast cancer and at high risk.

**4.2. Secondary objectives:**

1. Evaluate residual tissue after a skin-sparing mastectomy.

2. Identify risk factors for the presence of residual tissue after skin-sparing mastectomy.

3. Identify the factors related to satisfaction and quality of life after prepectoral reconstruction.

4. Identify the factors related to cosmetic sequelae after prepectoral reconstruction.

**5. Type of study**

Observational and prospective study

**6. Patients and method**

**6.1. Scope of study.**

The study will be carried out in patients diagnosed and treated in the Breast Unit of the Complejo Hospitalario Universitario de A Coruña.

**6.2. Study period.**

After the approval of the project by the Research Ethics Committee, the data will be collected. The study establishes a first evaluation of the patient at 12-18 months after surgery and a second evaluation at 5 years. It is estimated that the preliminary data are published 2-3 years after the start of the study and the final data after 6-7 years.

**6.3. Selection and withdrawal of patients:**

The study population includes two groups of patients:

a. Women at high risk for breast cancer. This group is made up of women evaluated in a high-risk consultation and whose risk reduction mastectomy has been approved by the tumor committee of the Hospital's Breast Unit. Patients must meet at least one of the following criteria:

- High-risk diagnosis for belonging to the hereditary syndrome for breast and ovarian cancer, either due to a demonstrated genetic mutation or due to family history.

- High-risk diagnosis due to the presence of high-risk histological lesions (atypical hyperplasia, ductal carcinoma in situ, lobular carcinoma in situ) associated with a family history.

- Previous diagnosis of breast carcinoma that during follow-up has been diagnosed at high risk for breast cancer.

The following patients were excluded from the study:

- Impossibility to carry out an MRI during follow-up (obesity, claustrophobia, etc).

- Impossibility of completing the BREAST-Q ™ questionnaire due to cognitive alterations of the patient or death.

- Refusal of the patient to participate in the study.

b. Women with breast carcinoma. This group is made up of patients with a diagnosis of breast carcinoma who require a mastectomy as surgical treatment. Patients must meet the following criteria:

- Histological diagnosis of carcinoma in situ or infiltrating breast, either primary or metachronous.

- Performance of a skin or skin-nipple sparing mastectomy.

- Carrying out an immediate reconstruction by placing an implant in a prepectoral position.

The following clinical situations are excluded from the study:

- Sarcomas of the breast.

- Benign breast tumors.

- Prepectoral reconstruction with expansion.

- Impossibility to carry out an MRI during follow-up (obesity, claustrophobia, etc).

- Impossibility of completing the BREAST-Q ™ questionnaire due to cognitive alterations in the patient.

- Refusal of the patient to participate in the study.

**6.4. Surgical method**

a. Mastectomy technique. Breast removal will be carried out by means of a mastectomy adapted to the patient's breast and optimizing the preservation of its original elements (inframammary groove, skin envelope, fat transitions, areola-nipple complex) according to the anatomical and oncological possibilities. For this, they will use skin-sparing mastectomies type 1, 2, 3 and 4 of the Carlson classification (15) or mastectomies with skin and nipple preservation through inframammary incision or vertical pattern.

b. Reconstruction technique. The reconstruction will be performed by placing a silicone implant in the prepectoral position. The silicone implant can be covered with mesh (biological or synthetic) or with polyurethane foam.

**6.5. Study measurements**

This study requires three study elements to achieve its objectives:

- MRI of the breast. From a clinical point of view, this diagnostic test is necessary for the follow-up of patients with breast reconstruction for the early diagnosis of relapses and deterioration of the breast implant. This study will use the first MRI during the postoperative period, between 12 and 18 months, to evaluate the residual tissue after mastectomy, in addition to those already mentioned above.

- BreastQ Questionnaire. This questionnaire is aimed at evaluating the satisfaction and quality of life reported by the patient through the use of the breast reconstruction module. The questionnaires used are the intellectual property of Columbia University (New York, USA), which grants their use for clinical research free of charge. This questionnaire is presented in two formats: the preoperative format, which will be delivered to the patient before the surgical intervention, and the postoperative questionnaire that will be delivered 12-18 months after said intervention. Subsequently, a second postoperative evaluation will be carried out 5 years after the surgical intervention.

- Iconography of the patient. The assessment of cosmetic sequelae requires taking photographs of the patient's torso (from the sternal pin to the navel). A photograph will be taken prior to the intervention (frontal, right lateral and left lateral), another at 12-18 months and finally at 5 years.

Simultaneously, the study will collect different variables from the patient's medical history:

- Epidemiological variables: age, weight, height, muscle mass index, smoking habit, fork-nipple distance, high risk assessment.

- Healthcare variables: stay, surgical time, complications, diagnosis-surgery delay, surgery-first adjuvant treatment delay, readmission, reintervention.

- Surgical variables: type of mastectomy, surgical piece weight, implant type, implant brand, lymph node staging.

- Histological variables: histological type, nuclear grade, final grade, lymphovascular invasion, intraductal component, lymph node involvement, number of affected lymph nodes.

- Oncological variables: primary systemic treatment, TNM, final staging, chemotherapy treatment, radiotherapy treatment, anti-hormonal treatment, biological treatment.

- Follow-up variables: residual tissue, residual tissue location, tumor recurrence, tumor recurrence location, deformity, location of the deformity, asymmetry.

- Satisfaction and quality of life variables: satisfaction with the breast, satisfaction with the information received, satisfaction with the surgeon, satisfaction with the nursing staff, satisfaction with the team, social well-being, sexual well-being, physical well-being.

**6.6. Sample size justification**

Assuming an incidence of local relapses of 5%, with a confidence level of 95% and a precision of 5%, and assuming 10% of possible losses during follow-up, the necessary sample size is estimated to be 81 patients.

**6.6. Statistic analysis.**

A descriptive analysis of the variables included in the study will be carried out. The quantitative variables will be expressed as mean and standard deviation, and the qualitative variables will be expressed as absolute value and percentage with the estimate of their 95% confidence interval. The comparison of means will be carried out by means of the Student's t test or the Mann-Whitney test and the Kruskal-Wallis test or ANOVA, as appropriate, after verifying normality with the Kolgomorov-Smirnov test. The association of qualitative variables will be estimated using the Chi-square statistic. The association between quantitative variables will be studied with the Pearson or Spearman correlation coefficient as appropriate. The study of the possible factors associated with the risk of invasion in these patients will be carried out using multivariate logistic regression, adjusting for those variables that are significant in the univariate or clinically relevant analysis. The risk of invasion will be expressed in Odds Ratio (OR), accompanied by its corresponding 95% confidence interval. Statistical analysis will be performed using version 24 of the IBM SPSS statistical program and epidat 4.1.

**7. Ethical-legal aspects**

The development of our research will be carried out respecting at all times the ethical precepts established in the Declaration of Helsinki of the World Medical Association 1964 and the Convention on human rights and biomedicine, made in Oviedo on April 4, 1997 and the regulations current in the field of health, research and protection of personal data, both European, state and regional, applicable to our study.

Researchers are familiar with the protocol and with data collection. Essential documents will be kept to demonstrate the validity of the study and the integrity of the data collected. The master files will be established at the beginning of the study, will be kept during its execution and will be kept in accordance with the applicable regulations. The Research Ethics Committee (REC) will review all relevant study documentation in order to safeguard the rights, safety, and well-being of patients. The study will begin after obtaining authorization from the competent CEI.

The researchers participating in this study undertake that all data collected from the study subjects is separated from the personal identification data, guaranteeing the confidentiality of the research participants and respecting the European Data Protection Regulation and Spanish regulations (both state and regional) in terms of data protection, health and research, current and applicable to the specific case.

The data of the patients will be collected by the researchers in the Data Collection Notebook (CRD) specific to the study. The file that relates the identity of the participant with the code assigned for the study will be kept separately by the person responsible for recruiting the patient. Each CRD will be coded, protecting the identity of the patient, and will be analyzed after recruitment is completed. Only the research team and the health authorities, who have a duty to keep confidentiality, will have access to all the data collected for the study.

Only information that cannot be identified may be transmitted to third parties (the code file will not be sent, only non-identifiable data will be sent).

Once the study is finished, the data will be anonymized for future use, requesting express permission for it in the IC from the participants.

The research team undertakes that an informed consent document and the BreastQ questionnaires will be delivered by the surgeon, so that they are aware of what their participation implies, in the healthcare consultation to the patients before they agree to participate in the study, and guarantee at all times, as shown in the Principal Investigator Commitment document, compliance with current legislation and the rules of good practice regarding confidentiality and data protection.

**8. Financing**

This project lacks external funding.

**9. Commitment of the researcher**

Attached

**10. Publication commitment**

The preliminary results of the study will be published between 2 and 3 years after its initiation. The final results of the study will be published at 6-8 years of age once an appropriate follow-up is obtained in cancer patients. During the course of the study, the results generated to date will be presented at national and international scientific meetings. Any publication or abstract arising from this study requires the approval of the principal investigator before publication or presentation. The anonymity of the subjects participating in the study will be maintained at all times.

**11. Bibliography**

1. Atisha D, Alderman AK, Lowery JC, Kuhn LE, Davis J, Wilkins EG. Prospective analysis of long-term psychosocial outcomes in breast reconstruction: two-year postoperative results from the Michigan Breast Reconstruction Outcomes Study. Ann Surg. 2008; 247 (6): 1019-28.

2. Heneghan HM, Prichard RS, Lyons R, Regan PJ, Kelly JL, Malone C, et al. Quality of life after immediate breast reconstruction and skin-sparing mastectomy - a comparison with patients undergoing breast conserving surgery. Eur J Surg Oncol. 2011; 37 (11): 937-43.

3. Temple-Oberle C, Ayeni O, Webb C, Bettger-Hahn M, Mychailyshyn N. Shared decision-making: applying a person-centered approach to tailored breast reconstruction information provides high satisfaction across a variety of breast reconstruction options. J Surg Oncol. 2014; 110 (7): 796-800.

4. Wagner RD, Braun TL, Zhu H, Winocour S. A Systematic Review of Complications in Prepectoral Breast Reconstruction. J Plast Reconstr Aesthet Surg 2019 Jul; 72 (7): 1051-1059.

5. Steven Sigalove. Prepectoral Breast Reconstruction and Radiotherapy-A Closer Look. Gland Surg 2019 Feb; 8 (1): 67-74.

6. de Vita R, Buccheri EM, Villanucci A, Pozzi M. Breast Reconstruction Actualized in Nipple-sparing Mastectomy and Direct-to-implant, Prepectoral Polyurethane Positioning: Early Experience and Preliminary Results. Clin Breast Cancer 2019 Apr; 19 (2): e358-e363.

7. Jeffrey Kluger, Alice Park. The angelina effect. Time. May 27, 2013.

8. Builes Ramírez S, Acea Nebril B, García Novoa A, Cereijo C, Bouzón A, Mosquera Oses J. Evaluation of the preoperative perception of quality of life and satisfaction of women with breast cancer using the BREAST-Q ™ questionnaire. Cir Esp. 2019 Dec 2. pii: S0009-739X (19) 30327-6. doi: 10.1016 / j.ciresp.2019.10.004.

9. Acea-Nebril B, Cereijo-Garea C, García-Novoa A, Varela-Lamas C, Builes-Ramírez S, Bouzón-Alejandro A, Mosquera-Oses J. The role of oncoplastic breast reduction in the conservative management of breast cancer : Complications, survival, and quality of life. J Surg Oncol. 2017; 115 (6): 679–686. doi: 10.1002 / jso.24550.

10. Acea Nebril B, García Novoa A, Polidorio N, Cereijo Garea C, Bouzón Alejandro A, Mosquera Oses J. Extreme Oncoplasty: The Last Opportunity for Breast conservation-Analysis of Its Impact on Survival and Quality of Life. Breast J 2019, 25 (3), 535-536. DOI: 10.1111 / tbj.13267.

11. Acea-Nebril B, Cereijo-Garea C, García-Novoa A, Bouzon-Alejandro A, Mosquera-Oses J. Breast-Q 15 Prospective Study: Oncoplastic Breast Reduction Improve Quality of Live for Women With Macromastia. Breast J 2020 Apr 14. doi: 10.1111 / tbj.13836.

12. Acea-Nebril B, García-Novoa A, Cereijo-Garea C, Builes-Ramirez S, Bouzon-Alejandro A, Mosquera-Oses J. Single-Incision Approach for Breast-Conserving Surgery: Effectiveness, Complications and Quality of Life. Ann Surg Oncol 2019, 26 (8), 2466-2474.

13. Cereijo C, Pita-Fernández S, Acea Nebril B, García Novoa A. Predictive factors of satisfaction and quality of life after immediate breast reconstruction using the BREAST-Q. Journal of Clinical Nursing 2018 Apr; 27 (7-8): 1464-1474.

14. Acea B, Cereijo C, García A. Cosmetic sequelae after oncoplastic breast surgery. Classification and factors for its prevention. Spanish Surgery 2015; 93 (2): 75-8.

15. G W Carlson 1, J Bostwick 3rd, T M Styblo, B Moore, J T Bried, D R Murray, W C Wood. Skin-sparing Mastectomy. Oncologic and Reconstructive Considerations. Ann Surg 1997; 225 (5): 570-5.

**12. Information sheet for the adult participant**

**STUDY TITLE:** Prepectoral reconstruction after oncological mastectomy and risk reduction. Prospective study for the evaluation of your safety, satisfaction and quality of life.

**PROMOTER / RESEARCHER**: Benigno Acea Nebril - María Lourdes García Jiménez

**CENTER:** A Coruña University Hospital Complex

This document is intended to provide you with information about a research study in which you are invited to participate. This study is being carried out at the A Coruña University Hospital Complex and was approved by the A Coruña-Ferrol Research Ethics Committee

If you decide to participate in it, you should receive personalized information from the researcher, read this document first and ask all the questions you need to understand the details about it. If you wish, you can bring the document, consult it with other people, and take the necessary time to decide to participate or not.

Participation in this study is completely voluntary. You can decide not to participate or, if you agree to do so, change your mind by withdrawing your consent at any time without giving explanations. We assure you that this decision will not affect your relationship with your doctor or the healthcare to which you are entitled.

**What is the purpose of the study?**

To determine the oncological safety, satisfaction and quality of life in patients with immediate breast reconstruction using a pre-pectoral technique with a polyurethane prosthesis.

**Why are you offering to participate to me?**

You are invited to participate because you have been diagnosed with breast carcinoma or have a high family risk for the development of a breast carcinoma, and in your case, it is intended to perform an immediate reconstruction with a prosthesis in a pre-pectoral position.

At least 81 people are expected to participate in this study.

**What does my participation consist of?**

The data collected in your medical history will be consulted during the process related to breast cancer (family and personal history, radiological studies, results of biopsies and surgical). It will also be requested to fill out a questionnaire about your body satisfaction before and after breast surgery, and pre and post-surgical photographic documentation will be collected only from the torso area (at no time will the facial area be included, so it will not be identification possible) for the assessment of the cosmetic result.

Your participation will have a total estimated duration of 5 -10 minutes or the time you need to cover both questionnaires.

**What inconveniences or inconveniences does my participation have?**

Their participation does not imply additional discomfort to those of normal clinical practice.

**Will I get any benefits for participating?**

You are not expected to benefit directly from participating in the study. The research aims to know the oncological safety, satisfaction and quality of life with pre-pectoral breast reconstruction. This information may be of use to other people in the future.

**Will I receive the information obtained from the study?**

If you wish, a summary of the study results will be provided to you.

**Will the results of this study be published?**

The results of this study will be sent to scientific publications for their dissemination, but no data that could lead to the identification of the participants will be transmitted.

**How will the confidentiality of my data be protected?**

The obtaining, treatment, conservation, communication and transfer of your data will be done in accordance with the provisions of the General Data Protection Regulation (EU Regulation 2016-679 of the European Parliament and of the Council, of April 27, 2016) and the regulations Spanish on protection of personal data in force.

The institution in which this research is carried out is responsible for the processing of your data, being able to contact the Data Protection Delegate through the following email: Delegado.Proteccion.Datos@sergas.es.

The data necessary to carry out this study will be collected and kept in a pseudonymized way (Coded), pseudonymization is the processing of personal data in such a way that they cannot be attributed to an interested party without additional information being used. The pre and post-surgical photographs of the torso area collected for cosmetic evaluation will be treated and preserved in the same way as the data, that is, coded. In this study, only the research team will know the code that will allow them to know their identity.

The regulations that regulate the treatment of personal data, grant you the right to access your data, oppose, correct, cancel, limit its treatment, restrict or request the suppression of the same. You can also request a copy of these or have it sent to a third party (portability right).

To exercise these rights, you can contact the Center's Data Protection Delegate through the means of contact indicated above or the researchers of this study by e-mail: maria.lourdes.garcia.jimenez@sergas.es y / or Benigno.Acea.Nebril@sergas.es.

Likewise, you have the right to file a claim with the Spanish Data Protection Agency, when you consider that any of your rights have not been respected.

Only the research team and the health authorities, who have a duty to keep confidentiality, will have access to all the data collected by the study. Information that cannot be identified may be transmitted to third parties. In the event that any information is transmitted to other countries, it will be done with a level of data protection equivalent, at least, to that established by Spanish and European regulations.

At the end of the study, or the established legal term, the data and photographs collected will be deleted or kept anonymous for use in future research, according to what you choose on the consent signature sheet.

**Are there financial interests in this study?**

The researcher will not receive specific compensation for dedication to the study.

You will not be rewarded for participating.

Commercial products or patents may be derived from the study results. In this case, you will not participate in the originated economic benefits.

**How to contact the research team of this study?**

You can contact Dr. María Lourdes García Jiménez at the email maria.lourdes.garcia.jimenez@sergas.es and / or phone: 981178000, ext .: 296150.

**Thank you very much for your help.**

**13. DOCUMENT OF CONSENT FOR PARTICIPATION IN A RESEARCH STUDY**

**STUDY TITLE:** Prepectoral reconstruction after oncological mastectomy and risk reduction. Prospective study for the evaluation of your safety, satisfaction and quality of life.

**PROMOTER / RESEARCHER:** Benigno Acea Nebril - María Lourdes García Jiménez

**CENTER:** A Coruña University Hospital Complex

Me, ……………………………………………………………………………………………………………………………………………

a. I read the information sheet to the participant of the study mentioned above that was given to me, I was able to talk with: ………………………………………………………………… .. …… and ask all the questions about the study.

b. I understand that my participation is voluntary, and that I can withdraw from the study at any time, without having to give explanations and without this affecting my medical care.

c. I agree to my data being used under the conditions detailed in the participant information sheet.

d. I freely agree to participate in this study.

At the end of this study I accept that my photographs and data are:

___ Deleted

___ Retained anonymized for future use in other research

Signed: The participant, Signed: The researcher requesting consent

Name and Surname: Name and Surname:

Date: Date:

**14. DOCUMENT OF CONSENT BEFORE WITNESSES FOR PARTICIPATION IN A RESEARCH STUDY** (for cases where the participant cannot read / write)

*The impartial witness must identify himself and be someone outside the investigative team.*

**STUDY TITLE:** Prepectoral reconstruction after oncological mastectomy and risk reduction. Prospective study for the evaluation of your safety, satisfaction and quality of life.

**PROMOTER / RESEARCHER:** Benigno Acea Nebril - María Lourdes García Jiménez

**CENTER:** A Coruña University Hospital Complex

I, ……………………………… .. …………………………………………………………., As an impartial witness, affirm that in my presence:

a. Was read it to …………………………………………………………. about the study.

b. He understood that his participation is voluntary, and that he can withdraw from the study whenever he wants, without having to give explanations and without this affecting his medical care.

c. He agrees that his data be used in the conditions detailed in the information sheet for the participant.

d. You freely agree to participate in this study.

Upon completion of this study, you accept that your photographs and data are:

___ Deleted

___ Retained anonymized for future use in other research

Signed: The witness, Signed: The researcher requesting consent

Name and surname: Name and Surname:

Date: Date:
