# Supplementary material for: The PreQ-20 TRIAL: A prospective cohort study of the oncologic safety, quality of life and cosmetic outcomes of patients undergoing prepectoral breast reconstruction
Source: PLoS One. 2022 Jul 14;17(7):e0269426. doi: 10.1371/journal.pone.0269426 (PMC9282436; doi:10.1371/journal.pone.0269426)
Supplement: Attachment — Submitted filename: Comments to the Author 2.docx [file pone.0269426.s004.docx]

**Comments to the Author**

***Reviewer #1:***

*The authors faithfully responded to the reviewer's comments and revised the manuscript. However, it is necessary to change the "Breast Q-20 trial" to the "PreQ-20 trial" in the discussion of Abstract.*

We appreciate the reviewer's comment. We apologize for the mistake in the abstract discussion, and we correct it.

***Reviewer #2:***

*There are still some points that are difficult to agree with, but there seems to be no problem conducting clinical trials to assess the safety of the prepectoral implant based breast reconstruction as the current protocol.*

Thanks for the reviewer's comments. We also appreciate that the reviewer accepts the approach of the protocol.
